# Effect of Parental Age, Parity, and Pairing Approach on Reproduction in Strain 13/N Guinea Pigs (*Cavia porcellus*)

**DOI:** 10.3390/ani13050895

**Published:** 2023-03-01

**Authors:** Sarah C. Genzer, Timothy Flietstra, JoAnn D. Coleman-McCray, Cassandra Tansey, Stephen R. Welch, Jessica R. Spengler

**Affiliations:** 1 Comparative Medicine Branch, Division of Scientific Resources, Centers for Disease Control and Prevention, Atlanta, GA 30329, USA; 2 Viral Special Pathogens Branch, Division of High-Consequence Pathogens and Pathology, Centers for Disease Control and Prevention, Atlanta, GA 30329, USA

**Keywords:** guinea pig, breeding, reproduction, pairing strategy, age, sex ratio, litter size

## Abstract

**Simple Summary:**

Inbred strains of guinea pigs are a vital resource in the study of human disease and infection. However, maintenance of these colonies can be challenging due to lower reproductive fitness than outbred lines, and other strain-specific clinical considerations. Here, we report on the reproductive characteristics of strain 13/N guinea pigs, and investigate whether the choice of breeding parameters, including parental age, parity, and pairing approaches, can increase reproductive success in colonies.

**Abstract:**

Guinea pigs are important animal models for human disease, and both outbred and inbred lines are utilized in biomedical research. The optimal maintenance of guinea pig colonies, commercially and in research settings, relies on robust informed breeding programs, however, breeding data on specialized inbred strains are limited. Here, we investigated the effects of parental age, parity, and pairing approaches on mean total fetus count, percentage of female pups in the litter, and pup survival rate after 10 days in strain 13/N guinea pigs. Our analysis of colony breeding data indicates that the average litter size is 3.3 pups, with a 25.2% stillbirth rate, a failure-to-thrive outcome in 5.1% of pups, and a 10 day survival rate of 69.7%. The only variable to significantly affect the reproductive outcomes examined was parental age (*p* < 0.05). In comparison to adults, both juvenile and geriatric sows had lower total fetus counts; juvenile boars had a higher percentage of females in litters, and geriatric boars had a lower 10 day survival rate of pups. These studies provide valuable information regarding the reproductive characteristics of strain 13/N guinea pigs, and support a variety of breeding approaches without significant effects on breeding success.

## 1. Introduction

Guinea pigs are important animal models for human disease and have been used for approximately 200 years. Their first uses included being in a study measuring heat production during respiration [1], and they were later used as models for infectious diseases such as tuberculosis and diphtheria [2]. While the use of guinea pigs has declined since peaking in the 1960s, they continue to be utilized today to model pulmonary, sexually transmitted, ocular, aural, and gastrointestinal diseases of bacterial or viral origin [1]. Additionally, guinea pigs have hemomonochorial placentation, as do humans, as well as a gestational period that can easily be divided into distinct trimesters, supporting their role as a model for reproductive toxicology and other pregnancy-related health issues [3].

Strain 13/N guinea pigs, which also appear in the literature as strain 13 or 13N, were originally developed in the early 1900s by the United States Department of Agriculture (USDA) [4,5]. This inbred strain serves as a critical small animal model of human disease; strain 13/N guinea pigs are used to study various conditions, including autoimmune thyroiditis [6,7] and osteoarthritis [8,9,10], and are also used as models of disease for highly pathogenic human viruses. Strain 13/N guinea pigs are susceptible to severe and fatal disease following infection with pathogenic arenaviruses, including Lassa [11,12,13] and Lujo viruses [14], and recapitulate many characteristics of infection in humans. As such, these guinea pigs have been essential for investigations of viral pathogenesis, therapeutic screening, and vaccine development for these high-consequence, high-containment agents. In addition, differential susceptibilities to disease in inbred guinea pig strains allow for investigations of associated hereditary deficiencies and biomarkers [15,16]. Despite their notable utility, currently in the United States, there is only a modest number of strain 13/N guinea pig colonies in institutions, limiting the supply of these essential animals.

One of the challenges in maintaining colonies of strain 13/N guinea pigs is their breeding, since 13/N guinea pigs have smaller litter sizes [1] and slower growth rates when compared to outbred Hartley guinea pigs [17]. Maintaining a healthy animal population requires knowledge of herd health and efficient reproductive management. The literature generally describes the sexual maturity and breeding characteristics of guinea pigs as follows: male guinea pigs (boars) usually mature sexually and can mate as young as 2–3 months of age, and female guinea pigs (sows) typically mature sexually at 2 months of age (55 to 70 days). However, both males and females can mature and be fertile even earlier. For guinea pig breeding, most recommendations are for the sow’s first breeding to occur before 6–7 months of age. Sows that reach adulthood without a prior pregnancy may not be able to deliver their young normally, due to a normal stiffening of the fibrocartilaginous pubic symphysis, resulting in dystocia when the pubic symphysis fails to relax over the course of gestation. Females spontaneously ovulate, and can have estrous or fertile periods at any time of the year, but they are most common in the spring [18]. The estrous cycle length is 16 days (13–21 day range) [19]. A female is fertile for about 6 to 11 h, most often during night hours [18]. Female guinea pigs begin a new estrous cycle 2 to 10 h after giving birth [20]. The guinea pig gestation period is 59 to 72 days. The litter size ranges from one to eight pups, but a litter of two to four is more common. A female can give birth to up to five litters per year when continuously paired with a male [18,21].

Previous studies of guinea pigs have shown that various factors, including maternal weight at conception, seasonality, social stability, and concurrent lactation, can impact reproductive parameters, such as the litter size, sex ratio, and onset of puberty [22,23]. Similarly, maternal age, maternal weight, postpartum estrus breeding [24,25], concurrent lactation [26], and seasonality [27,28] have been reported to affect the litter size and pup sex ratio in other rodents. To help safeguard this valuable animal model, the aim of this study was twofold: to characterize breeding outcomes, such as the birthing interval, litter size, and still birth rate, and to analyze factors influencing reproduction results in strain 13/N guinea pigs, with a focus on litter size, litter sex ratio (examined as the percentage of females in each litter), and 10 day survival rate. The goal of these analyses was to identify parameters and methods to improve breeding efficiency, and to encourage positive reproductive outcomes in strain 13/N guinea pig colonies.

## 2. Materials and Methods

### 2.1. Ethics Statement

All animal procedures were approved by the CDC Institutional Animal Care and Use Committee and conducted in accordance with the *Guide for the Care and Use of Laboratory Animals* at an AAALAC International-accredited facility.

### 2.2. Guinea Pigs and Breeding Strategy

Strain 13/N male and female guinea pigs were housed under ABSL-2 conditions, in conventional racks or floor pens segregated by sex, in a climate-controlled laboratory with a 12:12 h light cycle. Animals were provided with ad libitum feed [Guinea Pig Diet 5025; LabDiet, www.labdiet.com (assessed on 21 January 2023), containing min 18% protein, min 4% crude fat, and max 16% crude fiber] and water, Timothy hay daily, and fresh vegetables three times a week. Over the study period, and for the length of time we have maintained the colony, the husbandry practices have remained largely consistent. The animals have been housed in the same facility (same climate and noise environment) with comparable feed and enrichment approaches.

When actively breeding, animals were kept as a male–female pair, in a trio, with 2 females to 1 male, or in a quad, with 3 females to 1 male. Active breeding groups may be maintained in standard rack caging or in smaller, partitioned floor pens that meet regulations for enclosure size. The colony was acquired in 2012 and began with 22 animals representing 10 sibling breeding groups from the subsequently depopulated colony at Iowa State University. Previously, the strain was maintained through continuous housing of male–female sibling pairs from birth; however, in our facility, juveniles are separated by sex at weaning, typically at 4–8 weeks old, and then selectively paired later. When animals were paired, males were removed after 6–8 weeks, or were co-housed with the female from the time of pairing through to parturition, to capitalize on the fertile post-partum estrus. Females were examined for pregnancy, beginning 4–6 weeks after pairing, by abdominal palpation and/or abdominal radiographs. Spontaneous abortions were recorded, as detected by the presence of significant vaginal bleeding and/or expulsion of fetal membranes, and were confirmed by veterinary staff.

### 2.3. Statistical Analyses

Kernel density estimation was used to assess the distribution of the ages for both sows and boars at delivery and the weights of sows by age at delivery. Breeding sows and boars were primarily young adults, although pairings also included juvenile and geriatric animals; a comparable proportion of age categories was observed between the sexes. ANOVA was used to analyze the differences in mean total fetus count, litter sex ratio, and survival rate after 10 days between juvenile (0 through 150 d), adult (151 through 900 d), and geriatric (older than 900 d) guinea pigs, based on the age, weight, and parity of the sow at delivery. The analyses were also conducted separately, considering boar age for each parameter. Differences were analyzed via ANOVA to account for other effects, and to determine which were significant. For all analyses, the probability that the null hypothesis was correct (*p*-value) was compared to α of 0.05.

## 3. Results

### 3.1. Data Collection and Summary of Breeding Characteristics

The following breeding data were collected when available for pairings between 2012 and 2021: the age of sow and boar at pairing and at the birth of offspring, breeding scheme type (male–female pair, trio, or quad), length and number of pairing attempts, number of pregnancies, pairing-to-birth interval (days), number of pups (including stillbirths and failure to thrives within 10 days of birth), and sex of the weaned pups. The dataset represents a total of 267 litters and 296 breeding events, involving 168 sows and 100 boars. Not all data were recorded for all pairings or pregnancies. Thus, only those pairings or pregnancies for which the parameter of interest was available were included in corresponding analyses.

**Figure 1:**
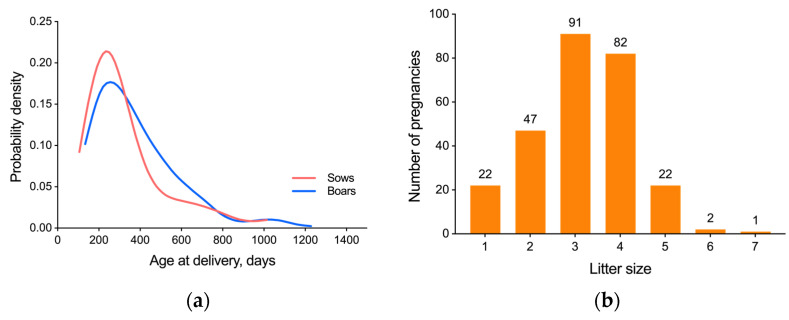
Overview of strain 13/N guinea pig colony, showing the parental breeding age and litter size. (**a**) Kernel Density Estimation for Age at Delivery for sows (*n* = 266) and boars (*n* = 260). (**b**) Distribution of number of fetuses in the dataset, from pregnancies yielding viable pups (*n* = 267).

Mean (median) age at primiparous parturition in the colony is 219.6 (213.5) days (*n* = 157). The oldest unassisted first birth was by a sow who was 388 days old (paired at 279 days of age). Amongst animals paired since birth, the mean (median) first birth occurs at 149 (136) days (*n* = 14). The youngest recorded birth was from a 105 day-old sow, and the youngest sire was 134 days old at time of birth. A small number of animals in the colony have been known to successfully breed after 3 years of age, with the oldest delivery overall in the colony being by an 1895 day-old sow (Figure 1a).

Litter size was normally distributed from one to seven pups, with most litters between three and four pups (Figure 1b). The average litter size was 3.3 pups, with a 25.2% stillbirth rate, with failure-to-thrive (not stillborn but do not survive 10 days) occurring in 5.1% of pups, and a 10 day survival rate of 69.7% (includes stillborn and failure-to-thrive). In this breeding cohort, there were 27 (10.1%) litters in which all pups were stillborn (range of one to five stillborn pups in litter; 81 pups total); whereas 87 (32.6%) litters had a mix of live and stillborn pups (range of one to four stillborn pups in litter, representing 16.7–80% of total litter size; 174 pups total). Additionally, there were 153 (57.3%) litters in which no pups were stillborn, with a range of one to five pups in the litter, 439 pups total. Overall, two pups were weaned per breeding attempt (590 pups weaned out of 296 total pairings). As biased sex ratios can reduce the size of the breeding population, tracking the operational sex ratio is critical for the conservation and reproductive management of both wild and laboratory populations [29]. The male-to-female birth ratio of strain 13/N guinea pigs was evenly split among weaned animals, with 296 females:294 male offspring from 181 weaned litters (leaving an additional 33 litters where no pups survived to weaning). The largest number of litters by a sow in a year was three (four sows), with most sows bearing one to two litters per year.

To further characterize breeding outcomes in our colony by age at delivery, the breeding population was investigated by using three broad age categories, as previously described for strain 13/N guinea pigs [30]: 0 through 150 d for juveniles, 151 through 900 d for adults, and older than 900 d for geriatric adults (Table 1 and Table 2).

### 3.2. Effects of Parental Age on Breeding Outcomes

**Figure 2:**
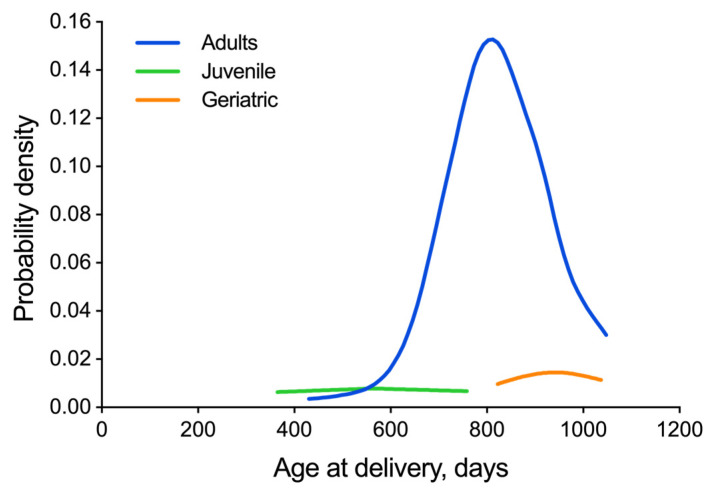
Kernel density estimation of guinea pig strain 13/N sow weight, by age at delivery and age group, as represented in the dataset. Sow weights followed a normal distribution with flatter curves, as expected, for juvenile (0 through 150 d, *n* = 19) and geriatric populations (older than 900 d, *n* = 7) due to the lower number of animals in those age categories relative to adults (151 through 900 d, *n* = 147).

To investigate the factors contributing to improved breeding outcomes, a series of analyses were conducted, examining the age at delivery (sow or boar), total fetus count, percentage of female pups in a litter, and survival rate after 10 days. ANOVA was used to analyze the differences in each parameter between adult guinea pigs and juvenile or geriatric ones, with category designation based on the age at delivery (Table 3 and Table 4). Factor effect Models were used to compare averages for juvenile and geriatric guinea pigs directly to those for adult animals. The analyses were conducted separately for sows and boars, for each measure. As age and weight are generally correlated, we also looked at weight separately as a potential confounding factor. Reproduction represents the most energetically demanding life history stage in mammalian females. Adequate and balanced dietary intakes of specific macronutrients are of major importance to ensure an appropriate energy supply for maintaining reproductive performances [31]. To eliminate potential bias in the age analysis due to a weight effect (as weight may reflect adequate or optimal energy balance), a kernel density estimation of sow weights was broken down by age category (scaled by pregnancies). The weight distribution of sows was normal, with a larger left tail partly attributed to juvenile pregnancies (Figure 2). No distributional difference was detected, neither in the adult population nor in juvenile or geriatric populations. We also evaluated the correlation of weight with total fetus count (0.0069, *p*-value = 0.41), to ensure that the age of the sow was the significant factor and not a matter of weight.

Overall, adult sows had a higher total fetus count (3.28, *n* = 240), on average, than either juvenile (2.21, *n* = 19) or geriatric (1.86, *n* = 7) sows, but the offspring of juvenile sows maintained a higher 10-day survival rate (0.86, *n* =19) than that of adults (0.71, *n* = 240). Geriatric sows had the lowest 10 day survival rate (0.42, *n* = 7) of the different age brackets. Adult sows had a significantly lower percentage of female pups (0.478, *n* = 211) than juvenile (0.696, *n* = 17) and geriatric (0.556, *n* = 3) sows. Overall, pairing with adult boars led to a higher total fetus count (3.24, *n* = 243), on average, than with either juvenile (2.57, *n* = 7) or geriatric (2.4, *n* = 10) boars. As seen with the sows, offspring of juvenile boars had the highest 10 day survival rate (0.79, *n* = 7), with those of adults (0.72, *n* = 243) being slightly lower and geriatric boars (0.45, *n* = 10) being significantly lower; the pairing with adult boars (0.489, *n* = 215) led to a significantly lower percentage of female pups than with juvenile boars (0.806, *n* = 6). The percentage of female pups from geriatric boars (0.183, *n* = 3) was even lower than seen with the adults, but did not reach statistical significance.

As the age of both the sows and boars had apparent impacts on the parameters of total fetus count, percentage of female pups in the litter, and 10 day survival rate, we sought to isolate the characteristics with the greatest effects by using ANOVA, including both sow and boar age categories. We started with the factors that were significant from the sow and boar individual analysis, and removed the factors that were now insignificant in the combined analysis in a stepwise manner, removing the least significant factor at each step until only the significant factors remained. When we added both sows and boars together, some of the factors went away. This phenomenon happens when factors are somewhat colinear with each other—in this case, adult sows and adult boars. Even though factors were significant when we looked at sows and boars separately, the factors we arrived at below are the ones that best account for the differences for each measure (Table 5). For total fetus count, values for both juvenile sows and geriatric sows remained significantly below the average count, while the geriatric boars were not a significant factor for the pooled analysis. Regarding the percentage of female pups, only the juvenile boars remained as a significant factor, having a 32% higher rate than the average. For the 10 day survival rate, geriatric boars were the only significant factor, having a 28% lower rate. These were the factors that were most responsible for the observed differences, but with larger sample sizes (particularly for geriatric guinea pigs), the excluded factors might be found to be significant.

### 3.3. Effects of Parity on Breeding Outcomes

Parity was reported to have a significant effect upon litter size in mice, particularly for litters conceived during postpartum estrus, but also for those conceived after lactational anestrus [24]. A study in small cohorts of mice (*n* = 18–22), with between 1 and 20 litters per individual, reported an initial increase in litter size after a primiparous litter, followed by a plateau period, and finally a period of linear decline, in which the mean number of young born decreased by one in each successive litter [25]. This decline was attributed to factors causing increased embryonic loss, rather than resulting from a diminished supply of eggs. To examine this phenomenon in our colony, breeding outcomes were analyzed based on sow parity. Sows in our dataset had a range of one to five total pregnancies (Table 4). Most sows had a single pregnancy, and the frequency of pregnancy for multiparous sows decreased as the total number of pregnancies increased (Table 6). Using these groupings, we looked at the impact of the number of pregnancies per sow on the total fetus count, percentage of female pups in a litter, and survival rate after 10 days. Because of the limited number of sows having three or more pregnancies, we looked at the averages across all pregnancies for the first, second, third, etc., and ANOVA was used to compare subsequent pregnancies to the initial one. In our sample cohort, the number of pregnancies was not found to have a significant impact on any of these measures (Table 6).

### 3.4. Effects of Pairing Strategies on Breeding Outcomes

Another component of this study was to evaluate the potential impacts of management strategies on breeding efficiency, including back-to-back breeding using the fertile post-partum estrus period, as well as pair vs. harem mating schemes. Our facility does not check for copulatory plugs as standard practice in our breeding program. Thus, we evaluate the “birthing interval”, the length of time from mate pairing to birth, as a substitution for true gestational length, keeping in mind the 16 day estrous cycle of guinea pigs. In back-to-back breeding, which takes advantage of the fertile post-partum estrus, an immediate pregnancy consists of a birth-to-birth interval equivocal to gestation length, with a median of 71 days. From our data, the birthing interval of back-to-back breeding is shorter, on average, than that of new pairings (81 days). We compared 45 pairings, where the breeding couple were not separated, to 201 new pairings (separated prior to pairing), using two sample t-tests to analyze the percentage of female pups, 10 day survival rate and total fetus count (Table 7). We found no significant differences between the two breeding strategies.

Another breeding strategy involves the pairing approaches, which include: (1) monogamous pairs (one male and 1onefemale), which is common for strains with good fecundity; (2) a mating trio (one male, two females), which maximizes cage space for breeding, and mothers assist in raising each other’s pups; (3) quad (harem) mating, which houses three females in a cage with one male (only one male per cage is allowed). For our analyses, breeding attempts were classified as male–female pairs (pair), two females to one male (trio), or three females paired with one boar (quad). We compared the trio and quad pairing strategies to the male–female pairs using ANOVA, in order to compare the birth interval, percentage of females in the litter, 10 day survival rate and total fetus count (Table 8). There were 236 total pairings with 112 pairs, 105 trios, and 19 quads. The different pairing strategies resulted in no significant differences between any of these variables.

## 4. Discussion

Here, we investigated the effects of a variety of parameters on breeding outcomes in a colony of inbred strain 13/N guinea pigs. Work with this lineage has provided critical advancements in therapeutic and vaccine development for human disease. These and other guinea pig inbred strains serve as highly translatable models for a wide variety of human biomedical research fields. To ensure the availability of these critical animals and to help optimize breeding outcomes in the colony, we characterized breeding trends, such as birthing interval, average litter size, and stillbirth rate. We further investigated several parameters that could be optimized in the future of the program, namely, the age of the parents, parity, and pairing strategies.

Overall, breeding characteristics were comparable to other guinea pig strains, including an average litter size that favored triplets, and a stillbirth rate that is just slightly higher (25.2%) than the 22% rate reported in long-haired guinea pigs (*Cavia aperea f. porcellus*) [32]. Of all variables investigated, parental age was the only factor associated with significantly different reproductive outcomes. In general, adult sows had a higher total fetus count than either juvenile or geriatric sows, but juvenile sows maintained a higher 10 day survival rate than adults, suggesting that there is a tradeoff consideration in age selection, and that both age categories can contribute to colony maintenance. Reproductive gain appears to wane in geriatric sows, which had the lowest 10 day survival rate of the age brackets. These data support efforts to breed younger age categories and remove geriatric sows from the breeding program, if feasible. Notably, our previously reported clinical data indicate that skeletal growth and maturation in strain 13/N guinea pigs continues through to at least 9 months old [30], and our breeding dataset confirms that the animals are capable of successful natural delivery with first breeding up to 9 months, one month beyond the 8 months of age typically recommended for guinea pigs, especially when closely monitored by veterinary staff and undergoing radiographic evaluation of the pubic symphysis, as well as subsequent close monitoring, throughout gestation, of symphysis relaxation prior to parturition.

We did not find a significant difference in any outcome measure associated with the number of pregnancies, in contrast to results reported in mice, indicating that litter size increases with subsequent pregnancies until plateauing and eventually declining [13]. However, as the decline in mice is not evident until birthing more than five litters, with most substantial effects seen after ten litters, it is possible that the changes previously observed may not have been captured in our dataset, given that the total number litters in our study population did not exceed five.

Pairing strategies can have effects on the characteristics of rodent litters. We found no significant differences between the two breeding strategies, regarding the timing of pairing and separation. As breeding success does not appear to be dependent on pairing, programs may select strategies that are preferred based on housing and resources, without compromising production outcomes. In our colony, while trio and harem pairing strategies have been employed for mating, generally, sows are separated at the time of delivery, to care for the pups. Interestingly, in mice, trio- and harem-raised weanling mice had significantly higher weaning weights than weanlings raised in a pair breeding configuration [33], suggesting that future efforts may consider maintaining these pairings post-partum, to optimize the health of the pups post-weaning.

## 5. Conclusions

Altogether, these studies provide valuable information regarding the reproductive characteristics of strain 13/N guinea pigs, establishing benchmarks for their litter size, stillbirth rate, and10 day survival rate. Additionally, the identification of important breeding parameters, as described herein, will aid in the propagation and management of these colonies. As strain 13/N guinea pigs are a vital research resource with utility in infectious disease research and the evaluation of medical countermeasures, the knowledge of factors that contribute to improved reproductive success is incredibly valuable and necessary.

## Figures and Tables

**Table 1:** Total fetus count and 10 day survival rate in strain 13/N guinea pig pregnancies by sow and boar age at delivery.

			Total Fetus Count		10 Day Survival Rate	
Age Category	*n*	# of Preg	Average	SD	Average	SD
**Sows**						
Juvenile	19	19	2.2	0.85	0.86	0.32
Adult	147	240	3.3	1.08	0.71	0.35
Geriatric	7	7	1.9	0.90	0.43	0.53
**Boars**						
Juvenile	7	7	2.6	1.62	0.79	0.39
Adult	84	243	3.2	1.08	0.72	0.35
Geriatric	6	10	2.4	1.07	0.45	0.50

*n*, indicates the number of individual sows and boars in each age category for the corresponding number of pregnancies; SD, standard deviation.

**Table 2:** Percentage of females in strain 13/N guinea pig litters by sow and boar age at delivery. Litter sex ratio was analyzed for a subset of the total pregnancies listed in Table 1. Both litter sex ratio data and sow age were known for 231 of 266 pregnancies, and litter sex ratio and boar age were known for 226 of 260 pregnancies.

			Percentage Female	
Age Category	*n*	# of Preg	Average	SD
**Sows**				
Juvenile	17	17	0.70	0.30
Adult	138	211	0.48	0.35
Geriatric	3	3	0.56	0.51
**Boars**				
Juvenile	6	6	0.81	0.22
Adult	82	215	0.50	0.35
Geriatric	5	5	0.18	0.29

*n*, indicates number of individual sows and boars in each age category for the corresponding number of pregnancies; SD, standard deviation.

**Table 3:** Analysis of effect of strain 13/N guinea pig sow age at delivery on breeding outcomes. ANOVA was used to analyze differences in mean total fetus count, percentage of female pups in the litter, and pup survival rate after 10 days for juvenile or geriatric guinea pigs, as compared to adult guinea pigs (t-Stat and *p*-value set as intercept). Age is based on the age of the parental sow at pup delivery.

Sow					
Variable	*n*	Estimate	Error	*t*-Stat	*p*-Value
**Total Fetus Count**					
Sow—Adult	240	3.28	−	−	−
Sow—Juvenile	19	−1.07	0.25	−4.22	0.000033 *
Sow—Geriatric	7	−1.43	0.41	−3.49	0.000566 *
**Percentage Female**					
Sow—Adult	211	0.47	−	−	−
Sow—Juvenile	17	+0.22	0.09	2.45	0.014912 *
Sow—Geriatric	3	+0.08	0.21	0.38	0.705116
**10 Day Survival Rate**					
Sow—Adult	240	0.71	−	−	−
Sow—Juvenile	19	+0.15	0.08	1.73	0.084573
Sow—Geriatric	7	−0.28	0.14	−2.08	0.038716 *

*, indicates values significant at *p* < 0.05; −, not applicable; estimate, the difference in the average for the respective group compared to adults; error, standard error of the corresponding estimator; *t*-Stat, *t*-statistic.

**Table 4:** Analysis of the effects of strain 13/N guinea pig boar age at delivery on breeding outcomes. ANOVA was used to analyze differences in mean total fetus count, percentage of female pups in the litter, and pup survival rate after 10 days for juvenile or geriatric guinea pigs as compared to adult guinea pigs. Age is based on the age of the parental boar at pup delivery.

Boar					
Variable	*n*	Estimate	Error	*t*-Stat	*p*-Value
**Total Fetus Count**					
Boar—Adult	243	3.24	−	−	−
Boar—Juvenile	7	−0.67	0.42	−1.60	0.111351 *
Boar—Geriatric	10	−0.84	0.35	−2.38	0.017905 *
**Percentage Female**					
Boar—Adult	215	0.49	−	−	−
Boar—Juvenile	6	+0.32	0.15	2.17	0.030858 *
Boar—Geriatric	3	−0.31	0.16	−1.93	0.055482
**10 Day Survival Rate**					
Boar—Adult	243	0.72	−	−	−
Boar—Juvenile	7	+0.06	0.14	0.45	0.650622
Boar—Geriatric	10	−0.27	0.12	−2.38	0.018261 *

*, indicates values that are significant at *p* < 0.05; −, not applicable; estimate, the difference in averages for the respective group compared to adults; error, standard error of the corresponding estimator; *t*-Stat, *t*-statistic.

**Table 5:** Final significant factors determined by stepwise regression in strain 13/N guinea pig pregnancies, represented by sow and boar age at delivery.

Variable	Estimate	Error	*t*-Stat	*p*-Value
**Total Fetus Count**				
Sow—Juvenile	−0.98	0.28	−3.55	0.00045 *
Sow—Geriatric	−1.38	0.58	−2.38	0.01783 *
**Percentage Female**				
Boar—Juvenile	0.32	0.15	2.21	0.0283 *
**10 Day Survival Rate**				
Boar—Geriatric	−0.28	0.11	−2.40	0.0173 *

*, indicates values significant at *p* < 0.05; estimate, the difference in average for respective group compared to adults; error, standard error of the corresponding estimator; *t*-Stat, *t*-statistic.

**Table 6:** The impact of the number of pregnancies of strain 13/N guinea pig sows on total fetus count, percentage of females in a litter, and survival rate after 10 days, as compared to first pregnancies.

Birth Count					
Preg #	# of Preg	Average	SD	*t*-Stat	*p*-Value
**1**	157	3.18	1.08	−	−
**2**	71	3.15	1.21	−0.15	0.884566
**3**	26	3.15	1.29	−0.10	0.918277
**4**	9	3.22	0.67	0.11	0.909614
**5**	3	3.00	1.00	−0.27	0.786132
**Percentage Female**					
**Preg #**	**# of Preg**	**Average**	**SD**	***t*** **-Stat**	***p*** **-Value**
**1**	157	0.48	0.34	−	−
**2**	71	0.50	0.37	0.41	0.682959
**3**	26	0.63	0.40	1.84	0.066368
**4**	9	0.37	0.36	−0.77	0.440866
**5**	3	0.33	0.58	−0.68	0.494708
**10 Day Survival Rate**					
**Preg #**	**# of Preg**	**Average**	**SD**	***t*** **-Stat**	***p*** **-Value**
**1**	157	0.73	0.34	−	−
**2**	71	0.68	0.38	−0.97	0.333474
**3**	26	0.74	0.38	0.11	0.913439
**4**	9	0.59	0.44	−1.13	0.259265
**5**	3	0.75	0.43	0.08	0.934618

−, not applicable; SD, standard deviation; *t*-Stat, *t*-statistic.

**Table 7:** Analysis of strain 13/N guinea pig breeding outcomes based on the separation of pairs following the breeding period. Breeding couples that were not separated (continuously paired) were compared to those separated prior to breeding (new pairing).

Variable	*n*	Estimate	Error	*t*-Stat	*p*-Value
**Total Fetus Count**					
Separated	45	3.19	−	−	−
Not Separated	201	0.09	0.18	0.52	0.604819
**Percentage Female**					
Separated	41	0.49	−	−	−
Not Separated	174	−0.01	0.06	−0.23	0.817538
**10 Day Survival Rate**					
Separated	45	0.67	−	−	−
Not Separated	201	0.11	0.06	1.90	0.058754

−, not applicable; *n*, number of pairing type for indicated analysis; estimate, the average difference for Not Separated compared to Separated pairings; error, standard error of the corresponding estimator; *t*-Stat, *t*-statistic.

**Table 8:** Analysis of the effect of strain 13/N guinea pig pairing approaches on breeding outcomes. Trio (one male, two females) and quad (one male, three females) pairing approaches were compared to pairs (one male, one female).

	Birth Interval				
Variable	*n*	Estimate	Error	*t*-Stat	*p*-Value
**Pair**	94	82.78	−	−	−
**Trio**	108	1.19	2.20	0.54	0.587681
**Quad**	13	7.68	4.50	1.71	0.089348
	**Total Fetus Count**				
**Variable**	***n***	**Estimate**	**Error**	***t*** **-Stat**	***p*** **-Value**
**Pair**	112	3.19	−	−	−
**Trio**	105	0.04	0.15	0.27	0.787221
**Quad**	19	0.13	0.28	0.46	0.644419
	**Percentage Female**				
**Variable**	***n***	**Estimate**	**Error**	***t*** **-Stat**	***p*** **-Value**
**Pair**	96	0.51	−	−	−
**Trio**	91	−0.04	0.05	−0.76	0.44849
**Quad**	18	−0.02	0.09	−0.26	0.796354
	**10 Day Survival Rate**				
**Variable**	***n***	**Estimate**	**Error**	***t*** **-Stat**	***p*** **-value**
**Pair**	112	0.72	−	−	−
**Trio**	105	−0.02	0.05	−0.44	0.661492
**Quad**	19	−0.02	0.09	−0.21	0.831265

−, not applicable; *n*, number of pairing events for indicated type; estimate, average difference for Trio or Quad, compared to the Pair, breeding strategies; error, standard error of the corresponding estimator; t-Stat, t-statistic.

## Data Availability

Data available upon request.
